# Supplementary material for: CLAVATA3 mediated simultaneous control of transcriptional and post-translational processes provides robustness to the WUSCHEL gradient
Source: Nat Commun. 2021 Nov 4;12:6361. doi: 10.1038/s41467-021-26586-0 (PMC8569176; doi:10.1038/s41467-021-26586-0)
Supplement: Supplementary file 1 — Supplementary Information [file 41467_2021_26586_MOESM1_ESM.docx]

Supplementary Materials for

CLAVATA3 mediated simultaneous control of transcriptional and post-translational processes provide robustness to the WUSCHEL gradient

Alexander Plong^1^, Kevin Rodriguez^1^, Mark Alber^2,3^, Weitao Chen^2,3^*, G. Venugopala Reddy^1,3^*

Correspondence to: [venug@ucr.edu](mailto:venug@ucr.edu) and [weitaoc@ucr.edu](mailto:weitaoc@ucr.edu)

**This document includes:**

Figs. S1 to S22

Tables S1 to S6

Captions for Movies S1 to S2

**Other Supplementary Materials for this manuscript include the following:**

Movies S1 to S2


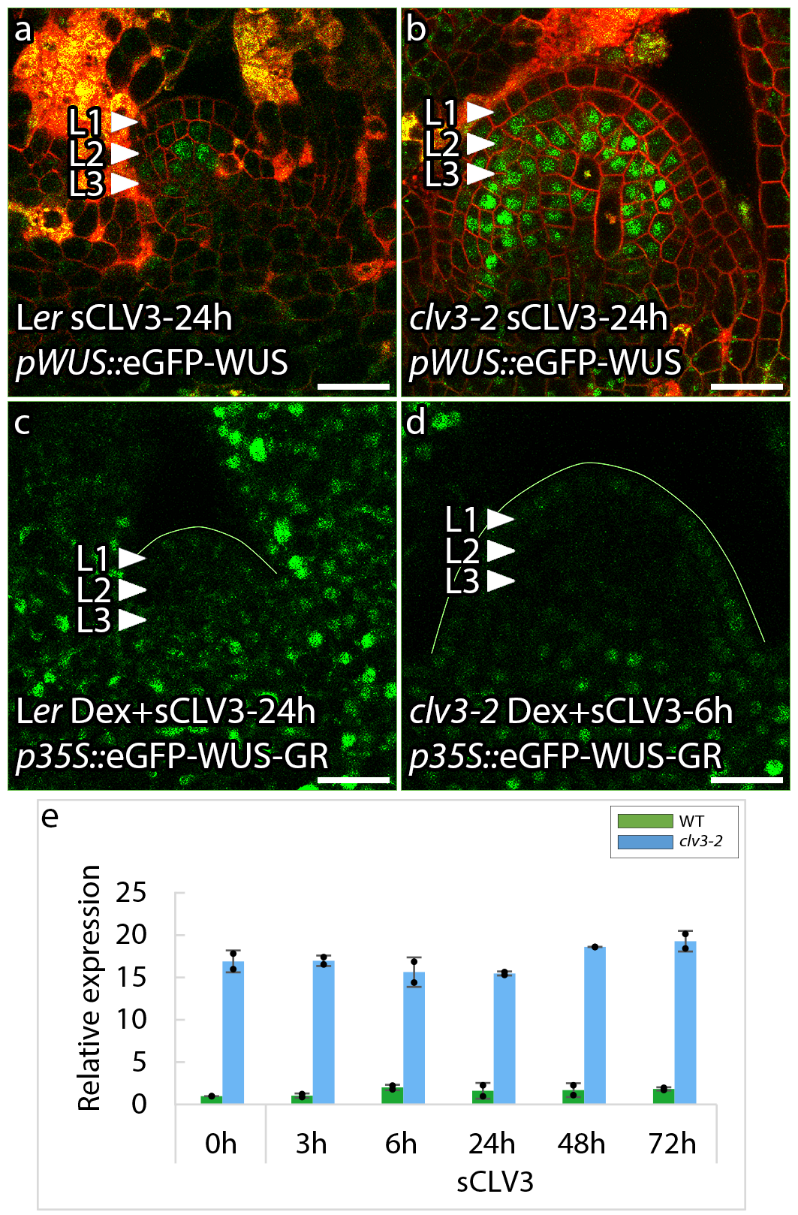


**Supplementary Figure 1. The scrambled CLV3 peptide (sCLV3) control treatments do not affect WUS protein or *WUS* transcript levels.** (a) *pWUS::*eGFP-WUS reporter response in 7-day old wild type plants to 1 μM sCLV3 treatment for 24 hours (n=7). (b) *pWUS::*eGFP-WUS reporter response in 7-day old *clv3-2* plants to 1 μM sCLV3 treatment for 24 hours (n=8). (c) *p35S::*eGFP-WUS-GR reporter response in 7-day old wild type to 24 hour Dex-induction co-treated with 1 μM sCLV3 (n=6). (d) *p35S::*eGFP-WUS-GR reporter response in 7-day old *clv3-2* to 6 hour Dex-induction co-treated with 1 μM sCLV3 (n=8). In (a-d) “n” represents the number of independently treated plants. eGFP (green) is overlaid on FM4-64 (red) plasma membrane stain. Scale bars = 20 μM. (e) Quantitative RT-PCR analysis was performed to determine the relative combined *WUS and eGFP-WUS* expression (mean ± s.d.) in 7-day old wild type and *clv3-2* mutants in response to 1 μM sCLV3 treatments for 3, 6, 24, 48, and 72 hours. Two biological replicates were used for each time point. *UBQ10* was used as a reference gene to normalize the data and relative changes in *WUS* expression were determined relative to the levels in wild type at 0 hours. Error bars represent the standard deviation (s.d).


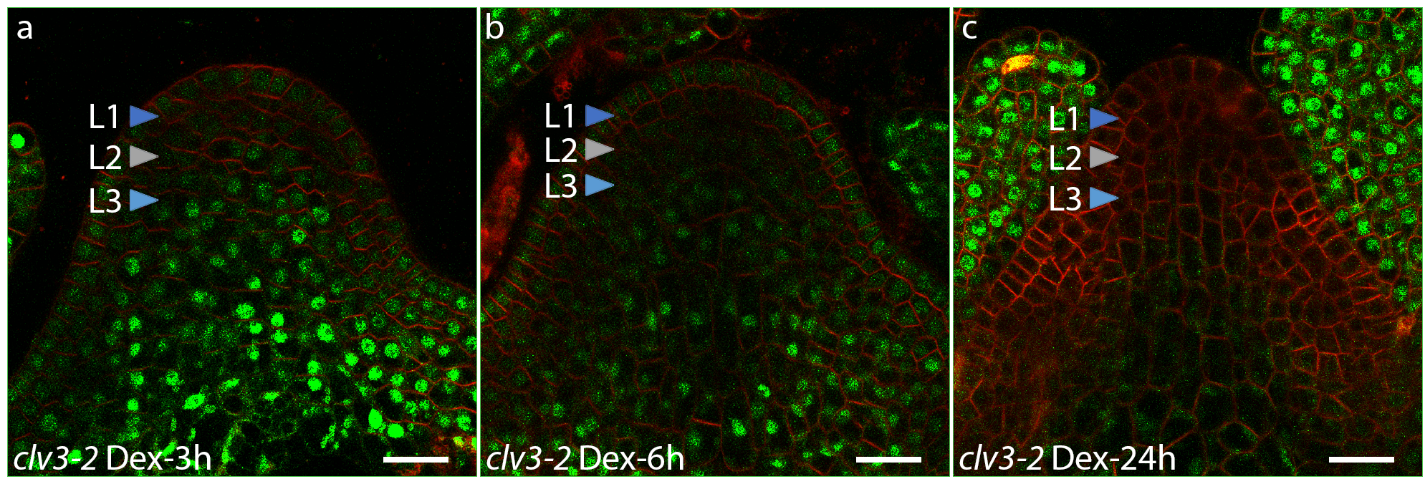


**Supplementary Figure 2. A drastic destabilization of eGFP-WUS-GR in *clv3-2* mutants upon Dex-induced nuclear translocation.** Dex-induction of *p35S::*eGFP-WUS-GR in *clv3-2* mutants at (a) 3 hrs (n=5), (b) 6 hrs (n=5), and (c) 24 hrs (n=5). eGFP (green) is overlaid on FM4-64 (red) plasma membrane stain. Scale bars = 20 μM. In all cases “n” represent the number of independently treated plants.


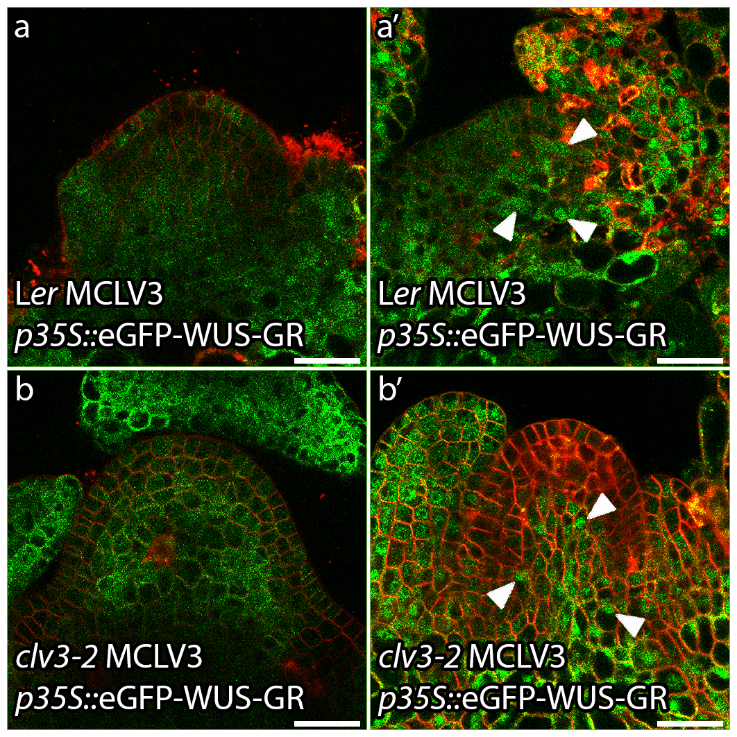


**Supplementary Figure 3. Occasional nuclear accumulation of *p35S::eGFP-WUS-GR* upon MCLV3 application before Dex treatment.** (a) Cytoplasmically localized *p35S::*eGFP-WUS-GR in wild type in response to 1 μM MCLV3 application (n=15). (b) Cytoplasmically localized *p35S::*eGFP-WUS-GR in *clv3-2* mutants in response 1 μM MCLV3 application (n=13). Arrows point to occasional nuclear accumulation before the Dex application in panels a’ (n=2) and b’ (n=2) which could be due to the leakiness of the GR fusion. eGFP (green) is overlaid on FM4-64 (red) plasma membrane stain. In all cases “n” represents the number of independently treated plants. Scale bars = 20 μM.


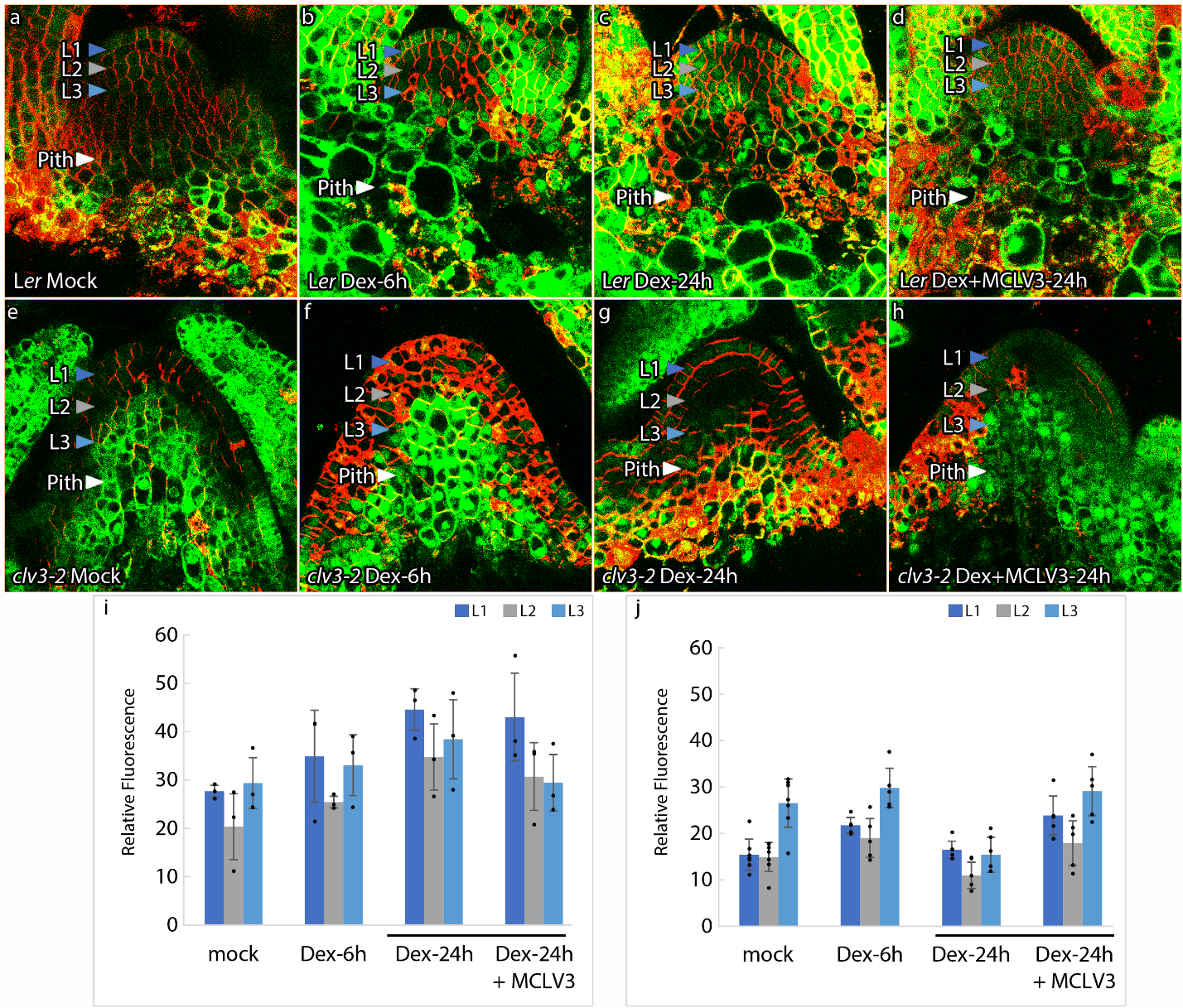


**Supplementary Figure 4. Stable nuclear accumulation of eGFP-GR upon its nuclear translocation in wild type and *clv3-2*.** *p35S::*eGFP-GR reporter response in 7-day old (a) uninduced (n=3), (b) short-term [6h] Dex-induction (n=3), (c) long-term [24h] Dex-induction (n=3), or (d) long-term Dex-induction [24h] co-treated with 1 μM MCLV3 (n=3) in wild type SAMs. *p35S::*eGFP-GR reporter response in 7-day old (e) uninduced (n=3), (f) short-term [6h] Dex-induction (n=4), (g) long-term [24h] Dex-induction (n=2), or (h) long-term Dex-induction [24h] co-treated with 1 μM MCLV3 (n=3) in *clv3-2* SAMs. In all cases “n” represents the number of independently treated plants. eGFP (green) is overlaid on FM4-64 (red) plasma membrane stain. Scale bars = 20 μM. (i-j) Quantification of the *p35S::*eGFP-GR fluorescence levels (mean ± s.d.) in (i) wild type or (j) *clv3-2* SAMs shown in a-h. Levels of eGFP-GR after 24 hrs Dex-induction were similar to Dex-induction co-treatment with MCLV3 for 24 hrs. The * represents p-value ≤ 0.05 in comparison to Dex treatment at 24 hours (Student’s two-tailed *t -*test). Error bars represent the standard deviation (s.d).


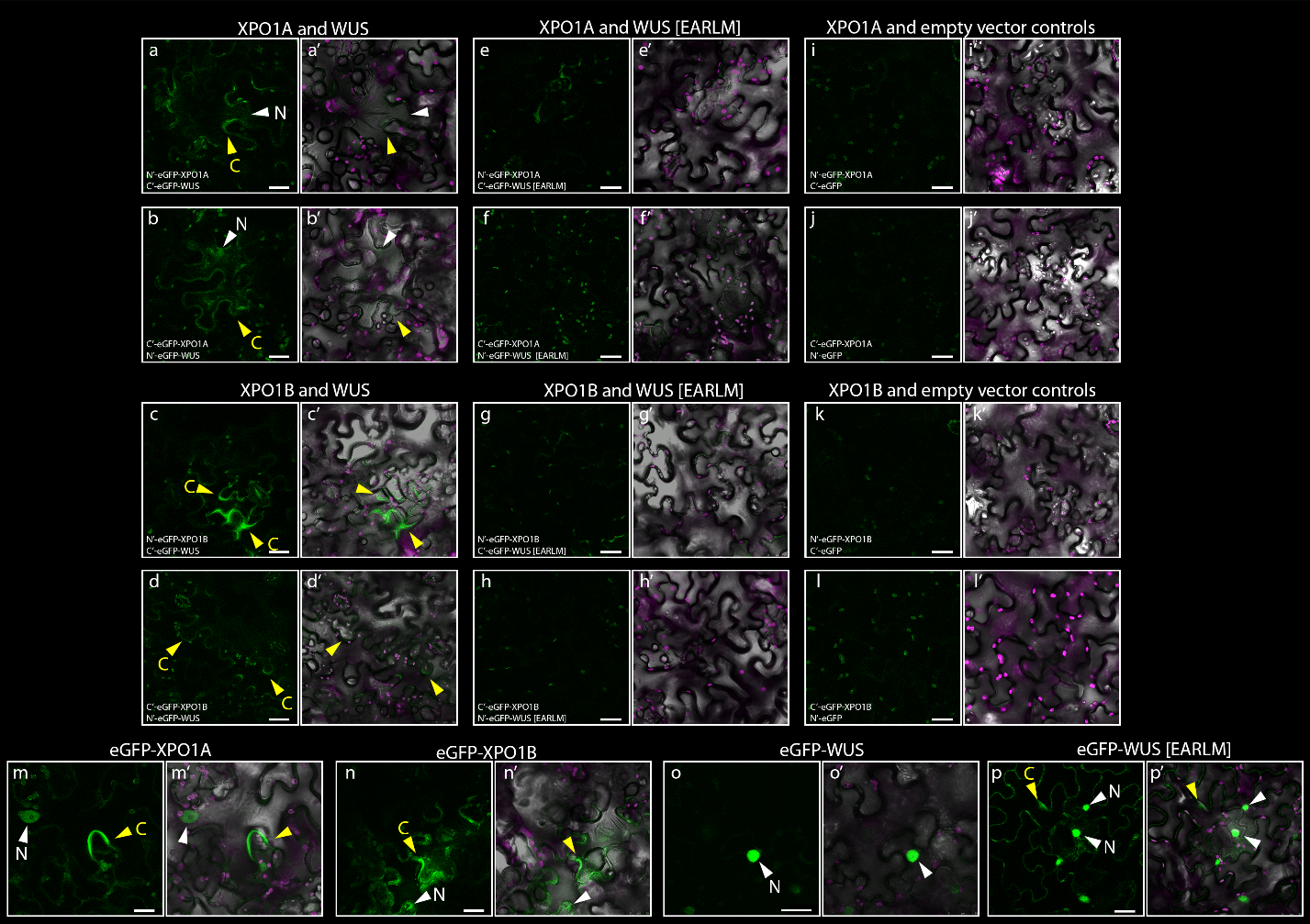


**Supplementary Figure 5. Bimolecular fluorescence complementation (BiFC) in *N. benthamiana* leaf cells showing the EAR-like domain dependent interaction between WUS with XPO1A and XPO1B.** Fluorescence complementation between (a-b) WUS and XPO1A (n=8, n=8) or (c-d) WUS and XPO1B (n=6, n=6) in nuclear and cytoplasmic compartments. Point mutations in the EAR-like domain of WUS abolishes fluorescence complementation between (e-f) WUS and XPO1A (n=5, n=4) or (g-h) WUS and XPO1B (n=7, n=6) revealing physical interaction is dependent on the leucine-rich repeat EAR-like domain. Lack of fluorescence with (i-j) XPO1A (n=8, n=4) or (k-l) XPO1B (n=6, n=5) with the empty vector controls. Accumulation of the positive controls for (m) eGFP-XPO1A (n=8), (n) eGFP-XPO1B (n=7), (o) eGFP-WUS (n=6), and (p) eGFP-WUS [EARLM] (n=8). Note the higher cytoplasmic accumulation of eGFP-WUS [EARLM] in transfected leaf cells. White arrows refer to nuclear localized eGFP signal and yellow arrows refer to cytoplasmically localized eGFP signal. In all cases “n” represents images from independent leaf sections. eGFP (green) and plastid autofluorescence (magenta) are overlaid on DIC in all [‘] panels. Scale bars = 20 μM.


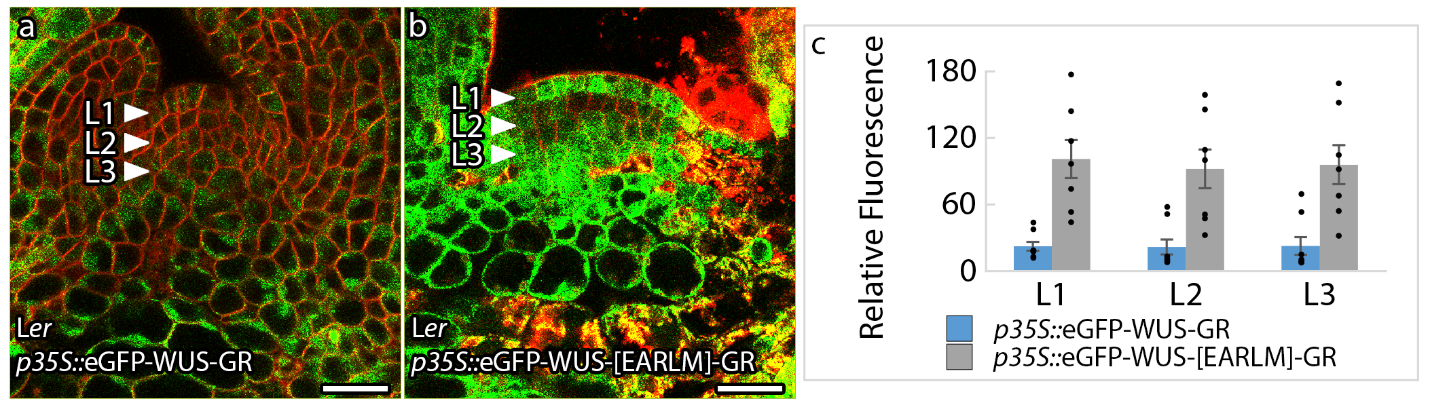


**Supplementary Figure 6. The EARlike domain mutants accumulate at higher levels in the cytoplasm.** (a) Uninduced wild type *p35S::*eGFP-WUS-GR reporter (n=8) and (b) the reporter with point mutations in the EAR-like domain (*p35S::*eGFP-WUS-GR [EARLM]) (n=7) in 7-day old wild type SAMs. In all cases “n” represents images from independent transgenic plants. (c) Quantification of fluorescence levels (mean ± s.d.) in SAMs of genotypes shown in a-b. Error bars represent the standard deviation (s.d.). eGFP (green) is overlaid on FM4-64 (red) plasma membrane stain. Scale bars = 20 μM.


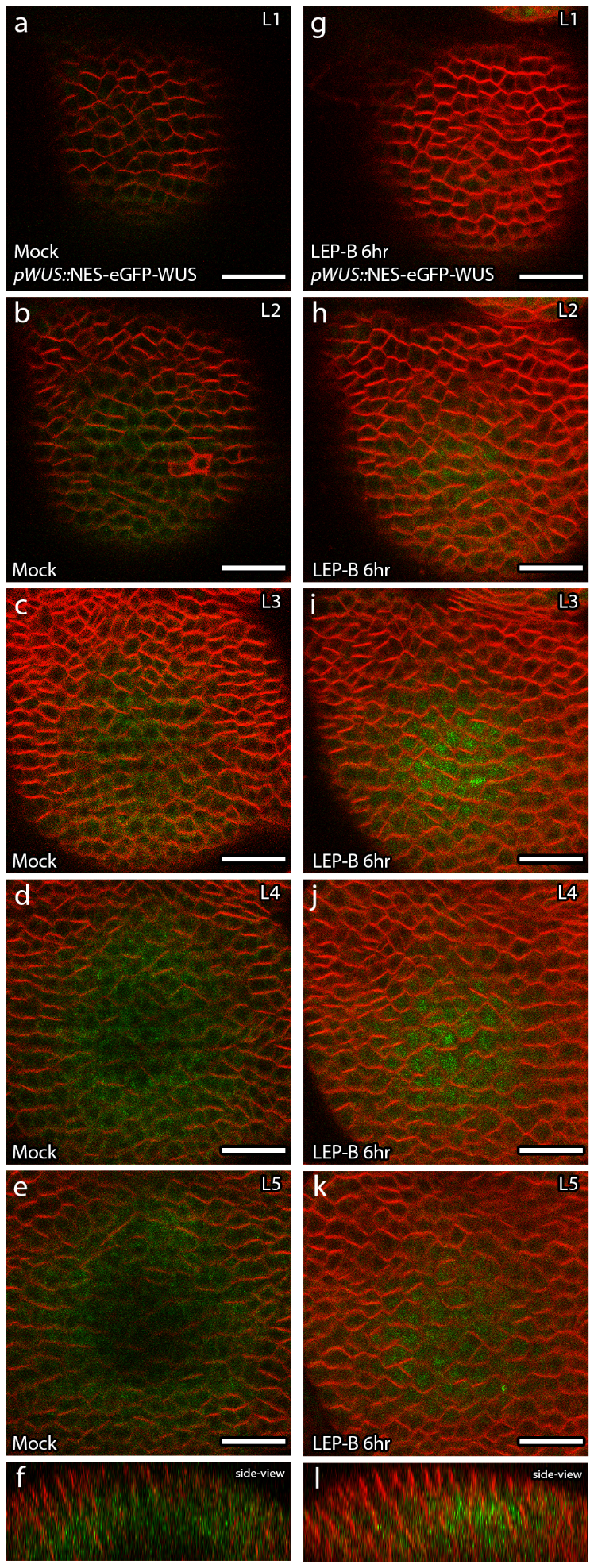


**Supplementary Figure 7. Nuclear accumulation of *pWUS::*NES-eGFP-WUS upon treatment with Leptomycin-B.** NES-tagged *pWUS::*NES-eGFP-WUS protein reporter when (a-f) untreated (n=6) in wild type SAM or (g-l) upon challenging with 20 nM LEP-B (n=6).

“n” represents the number of independently treated plants. Confocal cross sections cell layers L1-L5 are shown in (a-e) and (g-k). The side views are shown in (f) and (l). eGFP (green) is overlaid on FM4-64 (red) plasma membrane stain. Scale bars = 20 μM.

**
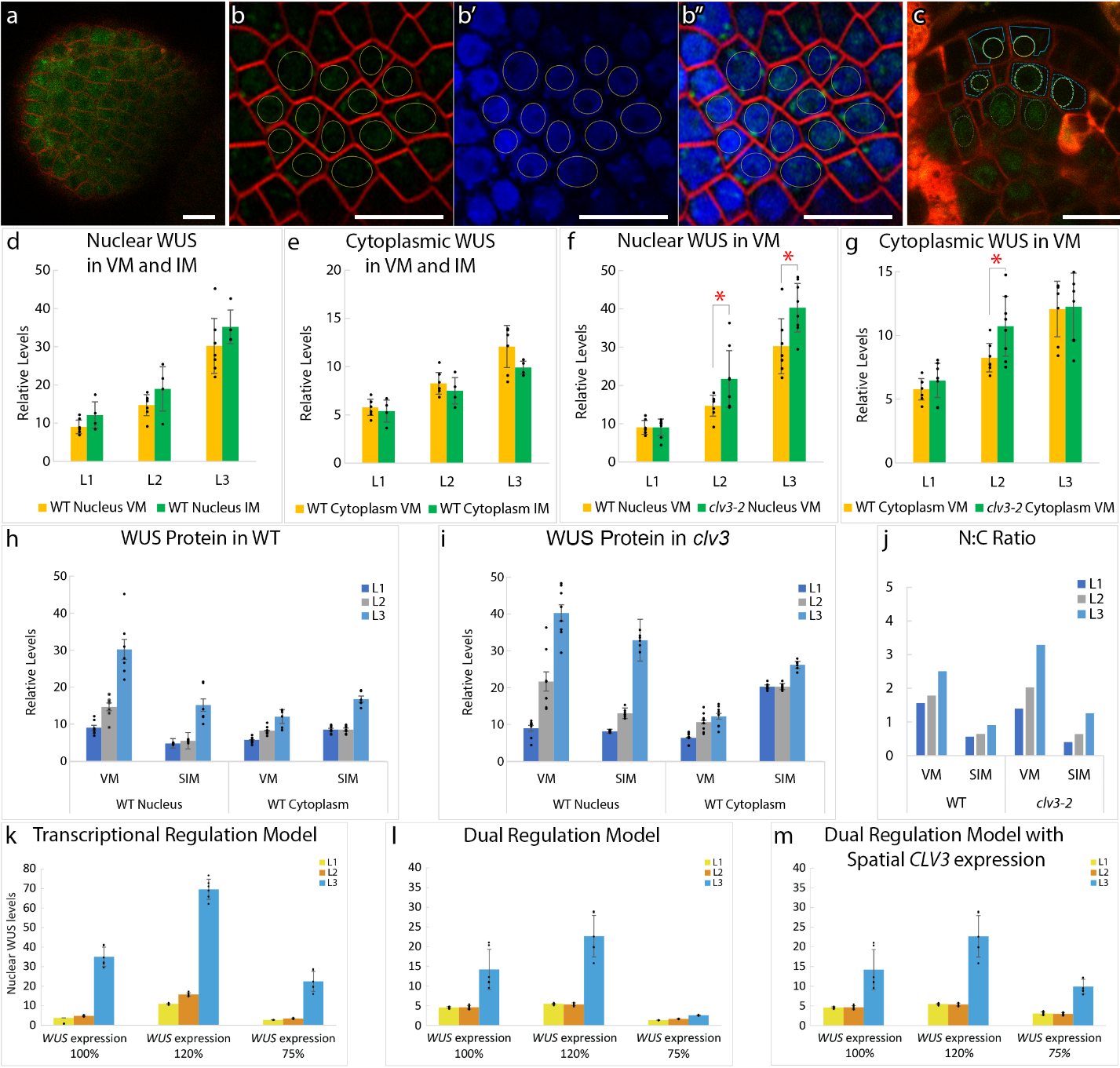
**

**Supplementary Figure 8. Quantification of *pWUS::*eGFP-WUS nuclear protein levels from experiments and simulations.** (a) eGFP channel fixation leads to autofluorescence in all cells. (b-c) Diagram of nuclear [yellow] and cytoplasmic [cyan] boundaries to quantify WUS levels from confocal micrographs in (b) the inflorescence meristem (IM) (n represents biological replicates [n=4]) or (c) the vegetative meristem (VM) (n represents biological replicates [n=7]). eGFP (green) is overlaid on FM4-64 (red) plasma membrane stain and DAPI (blue) DNA stain represents the nucleus. Scale bars = 10 μM. (d) Comparison of nuclear levels (mean ± s.d.) of eGFP-WUS in the wild type vegetative meristems (VM) [n=7] and inflorescence meristem [n=4]. (e) Comparison of cytoplasmic levels (mean ± s.d.) of eGFP-WUS in the wild type vegetative meristems [n=7] and inflorescence meristems [n=4]. (f) Comparison of nuclear levels (mean ± s.d.) of eGFP-WUS in the VM of wild type [n=7] and *clv3-2* mutants [n=8]. (g) Comparison of cytoplasmic levels of eGFP-WUS (mean ± s.d.) in the VM of wild type (n=7) and *clv3-2* [n=8]. The “n” represents the number of independent images used for fluorescence quantification. (h-i) Relative fluorescence levels of eGFP-WUS (mean ± s.d.) from VMs were compared to WUS levels (mean ± s.d.) from the simulations (SIM) in the nucleus and cytoplasm for each cell layer of (h) wild type (L1 [n=8], L2 [n=7] and L3 [n=6]), and (i) *clv3-2* mutants (L1 [n=8], L2 [n=7] and L3 [n=6]). The “n” in simulations represents the number of cells considered for quantification. (j) The nuclear:cytoplasmic ratios (N:C) of eGFP-WUS within each cell layer for wild type and *clv3-2* from h-i. (k-m) Quantification of simulated nuclear WUS levels upon perturbations of *WUS* expression rate in (k) the CLV3-mediated transcriptional regulation model, (L1 [n=8], L2 [n=7] and L3 [n=6], mean ± s.d.), (l) CLV3-mediated dual transcriptional and post-translational model (L1 [n=8], L2 [n=7] and L3 [n=6], mean ± s.d.), or (m) the dual regulation model with inclusion of spatial requirement of *CLV3* expression from the outer layers (L1 [n=8], L2 [n=7] and L3 [n=6], mean ± s.d.). The “n” in simulations represents the number of cells considered for quantification. The * represents p-value ≤ 0.05 (Student’s two-tailed *t-*test). Error bars represent the standard deviation (s.d.).


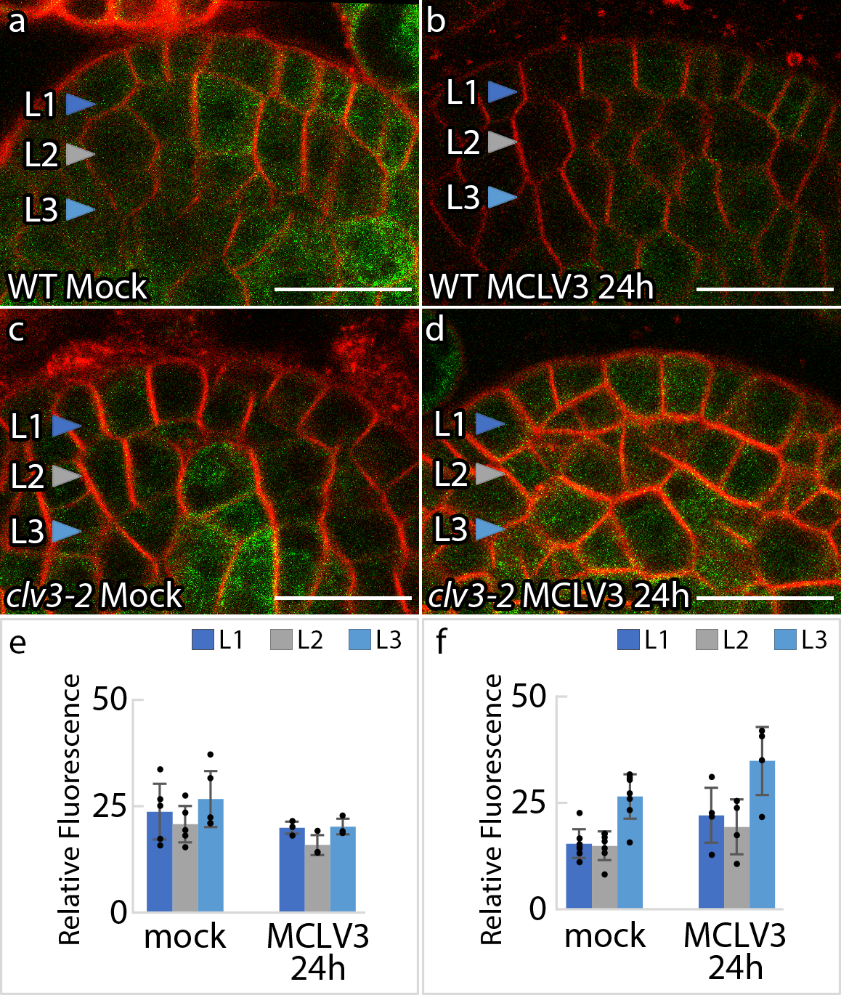


**Supplementary Figure 9. The cytoplasmic WUS is not stabilized by MCLV3 treatments.** Uninduced *p35S::*eGFP-WUS-GR reporter response in 7-day old wild type SAMs to (a) mock (n=5) or (b) 1 μM MCLV3 (n=3). Uninduced *p35S::*eGFP-WUS-GR reporter response in 7-day old *clv3-2* SAMs to (c) mock (n=7) or (d) 1 μM MCLV3 (n=4). In all cases “n” represents the number of independently treated plants. eGFP (green) is overlaid on FM4-64 (red) plasma membrane stain. Scale bars = 20 μM. (e-f) Quantification of *p35S::*eGFP-WUS-GR fluorescence levels (mean ± s.d.) in (e) wild type and in (f) *clv3-2* from SAMs shown in a-d. * represents p-value ≤ 0.05 in comparison to mock treatment from the same cell layer (Student’s two-tailed test). Error bars represent the standard deviation (s.d).


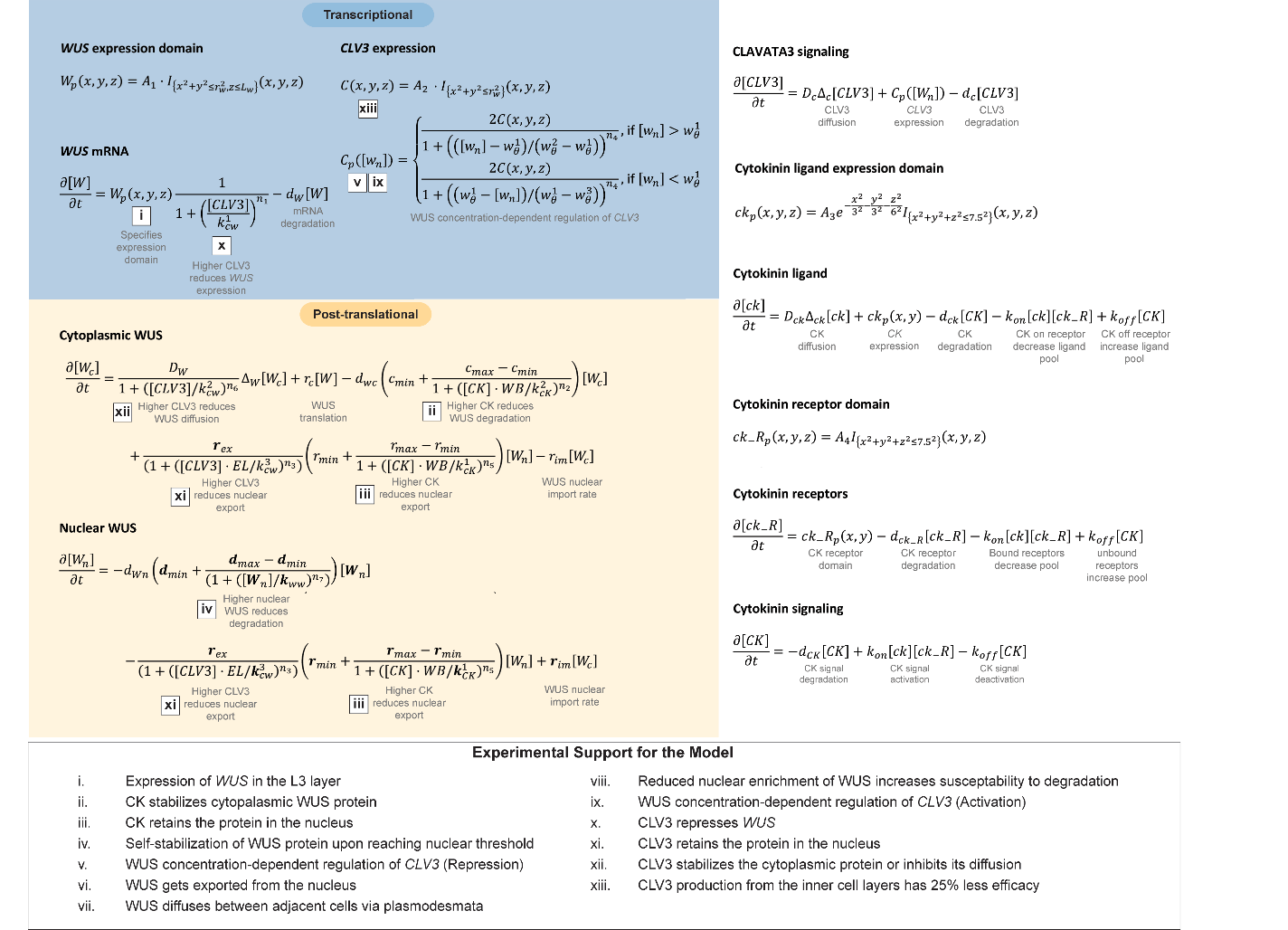


**Supplementary Figure 10. Computational model equations and biological support for the signal network.** Equations for biological chemical signals in the signaling network. The overall CLV3 signaling was modeled to simplify the binding process between the peptide and multiple CLV-type receptors distributed across the SAM, as per earlier study[^5,6^](https://paperpile.com/c/JSyvHi/3h0Q8+9q1mv). Regulation of WUS mRNA levels primarily regulated by CLV3-signaling which represses *WUS* expression, with post-translational feedback to promote nuclear enrichment by inhibiting the nuclear export of WUS and promoting cytoplasmic stability or limiting its diffusion. The CK ligand was modeled as a diffusive molecule throughout the SAM with the receptors localized only to the RM which promotes nuclear retention and cytoplasmic stability of the WUS protein[^16^](https://paperpile.com/c/JSyvHi/8v78n). This leads to lower nuclear WUS in the outer cell layers and higher nuclear WUS in the inner layers to activate and repress *CLV3*, respectively[^2,8,9^](https://paperpile.com/c/JSyvHi/thZ5x+Wyn5M+NhTDr).


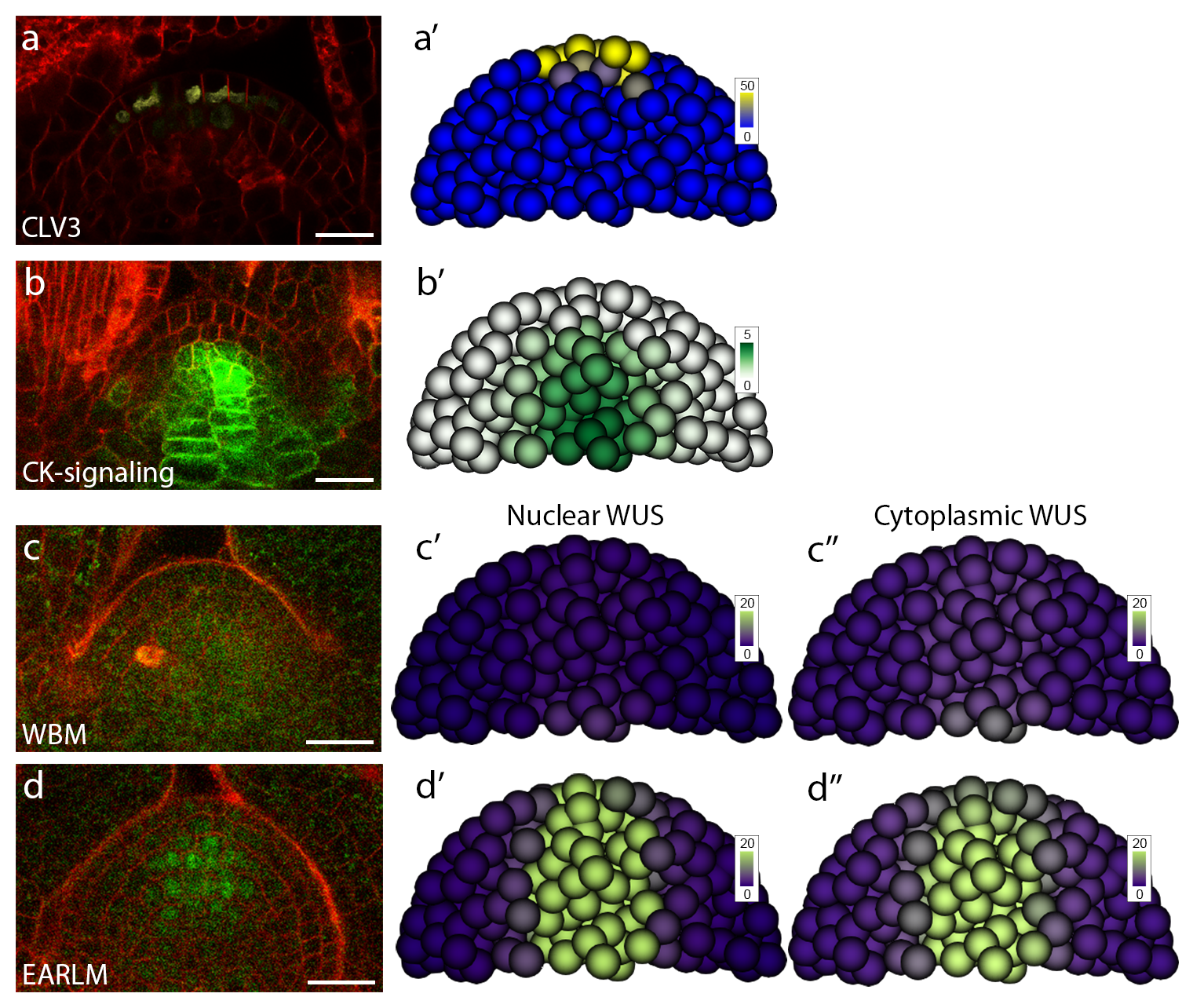


**Supplementary Figure 11. Benchmarking experimental observations to model simulations.** Comparison of signal distribution from experimental confocal micrographs to model simulation. The *CLV3* expression profile from (a) experimental images (*pCLV3::*H2B-mYFP reporter, n=9 ) and (a’) model simulation. The cytokinin signaling domain from (b) (*pTCSn::*mGFP-ER reporter) experimental images (n=10) and (b’) model simulation. Mutant *pWUS::*eGFP-WUS reporter distribution from experimental and model simulations of (c, c’, c’’) WUS-box motif mutants *pWUS::*eGFP-WUS [WBM] (n=12) and (d, d’, d’’) *pWUS::*eGFP-WUS [EARLM] (n=12) with perturbed nuclear retention and nuclear export, respectively [^8^](https://paperpile.com/c/JSyvHi/Wyn5M). In all cases n represent images from independent plants. eGFP (green) or mYFP (yellow) represents the mentioned signal and overlaid on FM4-64 (red) plasma membrane stain. Scale bars = 20 μM.


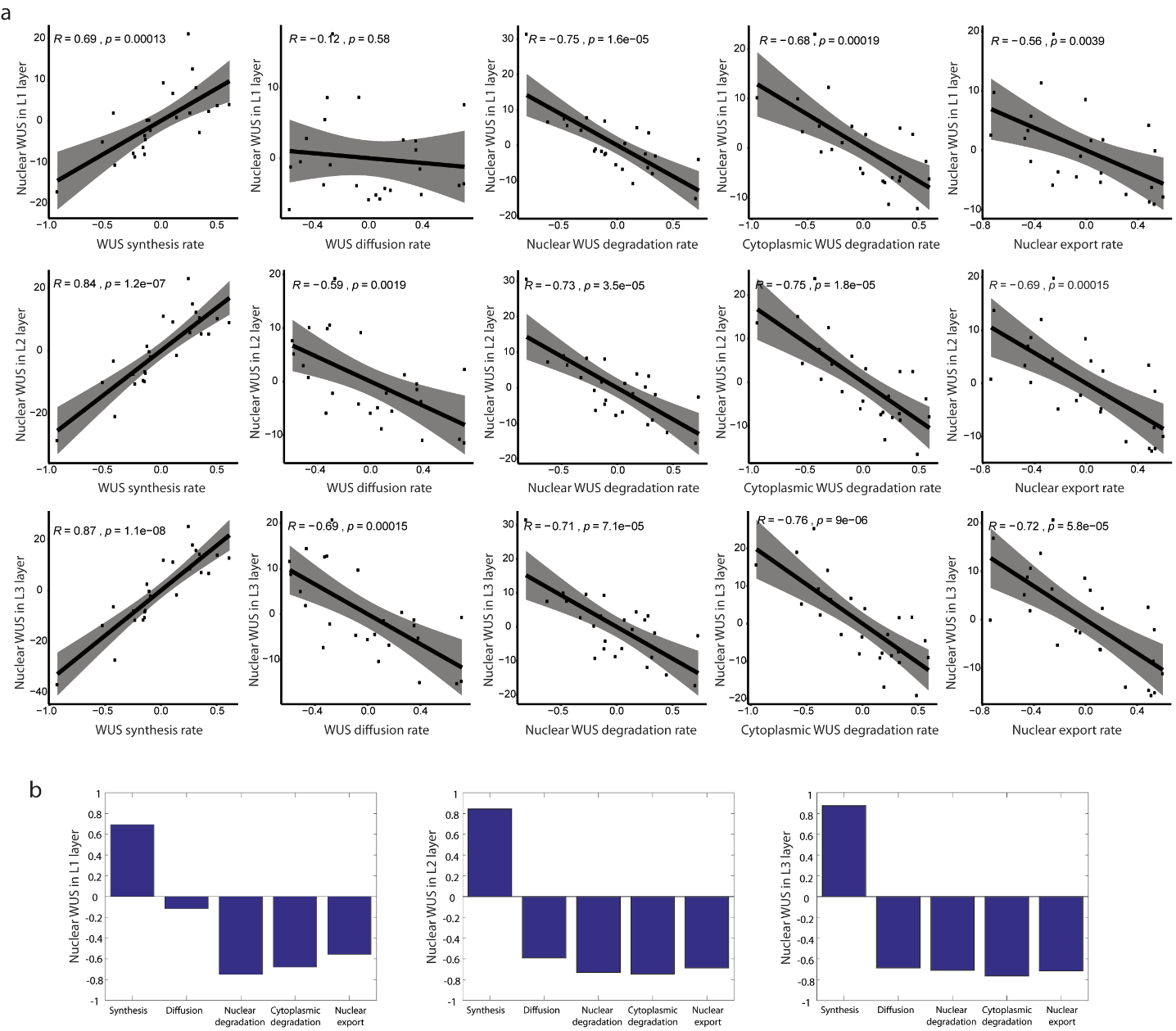


**Supplementary Figure 12. Local sensitivity analysis of the computational model.** (a) Correlation plots of sensitivity analysis of *WUS* synthesis rate, WUS diffusion rate, Nuclear WUS degradation rate, Cytoplasmic WUS degradation rate, or Nuclear export rate. Parameter perturbations are shown on x-axis for the three cell layers and the nuclear WUS protein concentration is shown on y-axis. The solid lines represent the linear regression lines of best fit and the grey areas represent the 95% confidence regions. (b) Bar plots showing the correlation (*R*) observed in sensitivity analysis in different cell layers. The x-axis represents perturbed parameters and y-axis represents sensitivity of the nuclear WUS protein concentration.

**
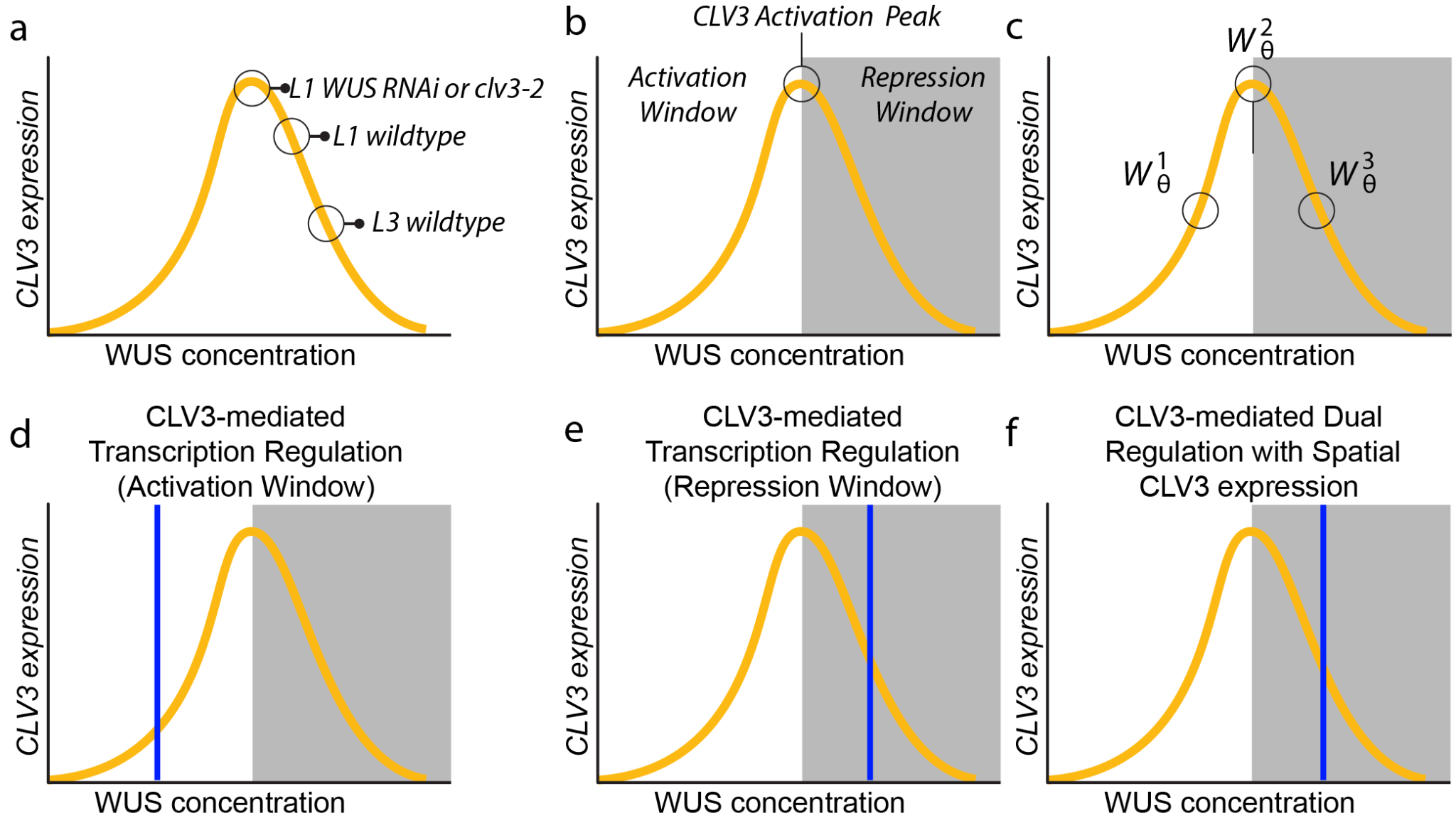
Supplementary Figure 13. *CLV3* expression in response to WUS concentration.** (a-f) Schematic diagrams showing *CLV3* expression window in response to WUS concentration. (a-c) The peak *CLV3* expression ($W_{\theta}^{2}$) has been observed in Layer 1 (L1) of *clv3-2* null mutants or upon partial downregulation of *WUS* transcripts achieved by transiently expressing an artificial microRNA (amiRNA) targeting *WUS*, suggesting that the *CLV3* expression in the L1 layer of wild type SAMs is under slightly repressive state. (c) Two representative points of decreased *CLV3* expression to 50% of the peak expression due to reduced activation, denoted by $W_{\theta}^{1}$, and due to increased repression, denoted by $W_{\theta}^{3}$. (d-f) WUS concentrations used in unperturbed simulations (WUS expression 100% in Supplementary Figures 15-18) at which *CLV3* is expressed in the L1 and L2 cell layers (blue vertical lines) within either the activation (d) or repression (e) window in CLV3-mediated transcriptional regulation model and within the repression window in CLV3-mediated dual regulation model with the spatial *CLV3* expression (f).


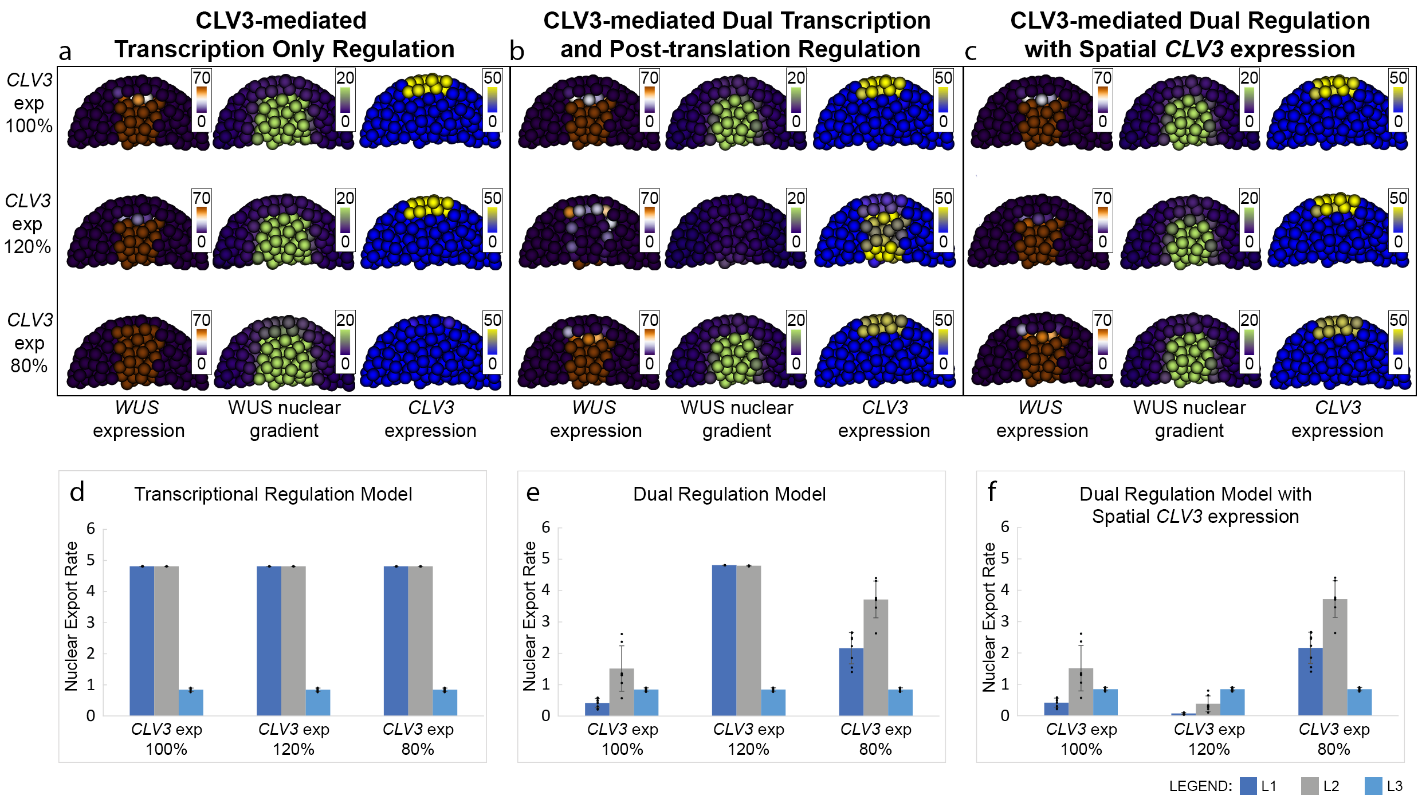


**Supplementary Figure 14. Dynamics of WUS-CLV3 feedback in simulations with CLV3-mediated transcriptional regulation, dual transcriptional & post-translational regulation, and dual regulation with spatial requirement of *CLV3* expression from the outer layers, upon perturbing *CLV3* expression rate**. *WUS* expression (brown), the WUS nuclear protein gradient (green), and *CLV3* expression (yellow) in the SAM upon perturbations in *CLV3* expression rate in simulated (a) CLV3-mediated transcriptional regulation model, (b) CLV3-mediated dual transcriptional and post-translational regulation model, and (c) CLV3-mediated dual regulation with the spatial requirement of *CLV3* expression from outer layers model. The nuclear export rates across the cell layers upon perturbation in *CLV3* expression rate in simulated (d) CLV3-mediated transcriptional regulation model (for simulations n represents the number of cells per layer L1 [n=8], L2 [n=7] and L3 [n=6], mean ±s.d.), (e) CLV3-mediated dual transcriptional and post-translational regulation model (for simulations n represents the number of cells per layer L1 [n=8], L2 [n=7] and L3 [n=6], mean ±s.d.), and (f) CLV3-mediated dual regulation with the spatial requirement of *CLV3* expression from outer layers model (for simulations n represents the number of cells per layer L1 [n=8], L2 [n=7] and L3 [n=6], mean ±s.d.). Error bars represent the standard deviation (s.d.). **
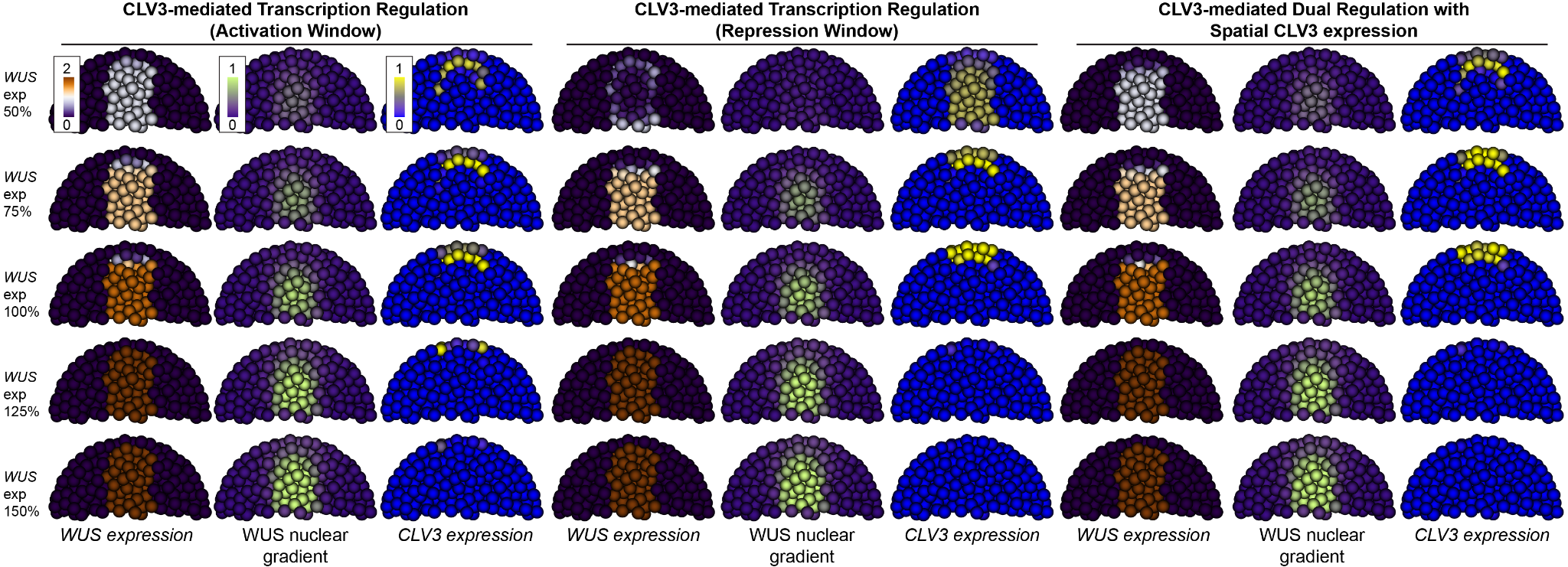
**

**Supplementary Figure 15. First additional parameter set to demonstrate the robustness of the *CLV3* expression domain improved by CLV3-mediated dual regulation with spatial *CLV3* expression.** In this parameter set, WUS mRNA $A_{1}=0.1715$, diffusion rate of WUS $D_{w}=0.0505$, degradation rate of $W_{n}$ $d_{wn}=0.0626$, degradation rate of $W_{c}$ $d_{wc}=0.0264$, and nuclear export rate $r_{ex}=1.2864$. CLV3-mediated transcriptional regulation maintains *CLV3* expression in L1 and L2 cell layers for lower WUS expression rates when *CLV3* is activated within the activation window of WUS concentration (left three columns). It fails to limit *CLV3* expression in L1 and L2 cell layers for lower *WUS* expression rates when *CLV3* is activated within the repression window of WUS concentration (middle three columns). The dual regulation with spatial *CLV3* expression can at least maintain *CLV3* expression in L1 and L2 cell layers for reduced *WUS* expression rates when *CLV3* is activated within the repression window of WUS concentration (right three columns).

**
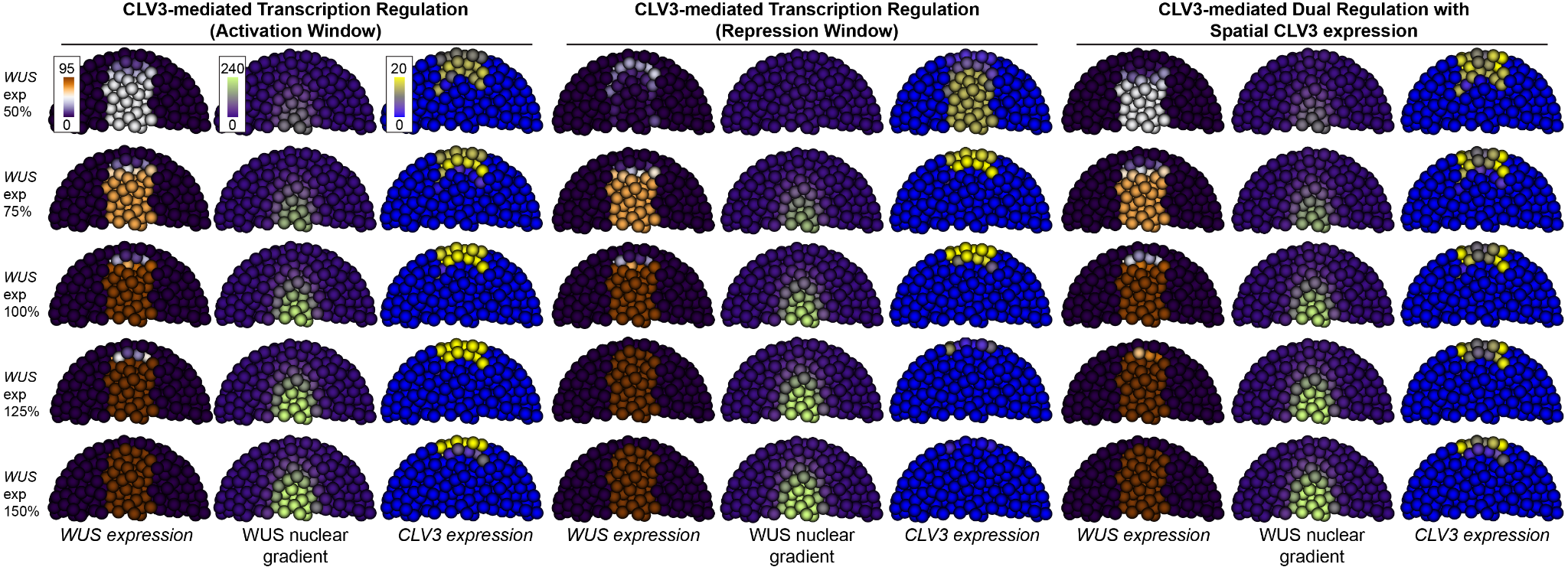
**

**Supplementary Figure 16. Second additional parameter set to demonstrate the robustness of the *CLV3* expression domain improved by CLV3-mediated dual regulation with the spatial *CLV3* expression.** In this parameter set, WUS mRNA $A_{1}=92.2240$, diffusion rate of WUS $D_{w}=1.0090$, degradation rate of $W_{n}$ $d_{wn}=0.0610$, degradation rate of $W_{c}$ $d_{wc}=1.5538$, and nuclear export rate $r_{ex}=1.0650$. CLV3-mediated transcriptional regulation maintains *CLV3* expression in L1 and L2 cell layers for both lower and higher *WUS* expression rates when *CLV3* is activated within the activation window of WUS concentration (left three columns). It fails to maintain *CLV3* expression in L1 and L2 cell layers for both lower and higher *WUS* expression rates when *CLV3* is activated within the repression window of WUS concentration (middle three columns). The dual regulation with the spatial *CLV3* expression can maintain *CLV3* expression in L1 and L2 cell layers for both reduced and increased *WUS* expression rates when *CLV3* is activated within the repression window of WUS concentration (right three columns).


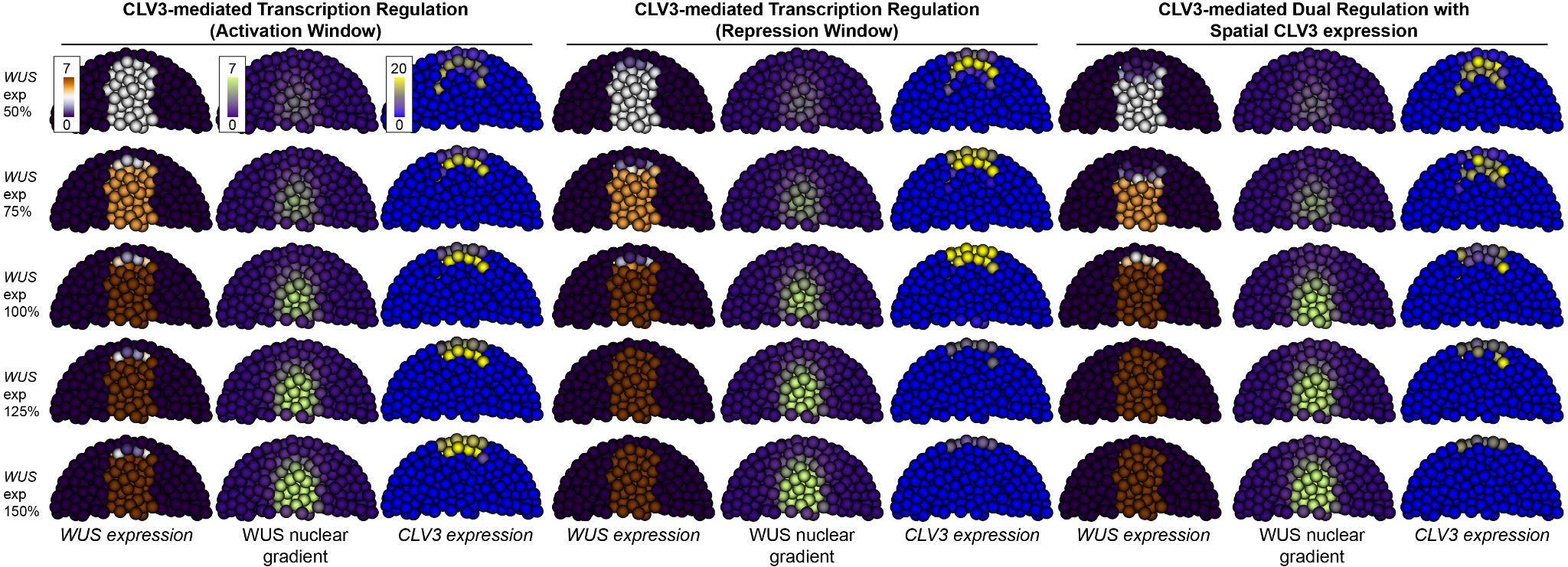


**Supplementary Figure 17. Third additional parameter set to demonstrate the robustness of the *CLV3* expression domain improved by CLV3-mediated dual regulation with the spatial *CLV3* expression.** In this parameter set, WUS mRNA $A_{1}=0.6978$, diffusion rate of WUS $D_{w}=0.0698$, degradation rate of $W_{n}$ $d_{wn}=0.0294$, degradation rate of $W_{c}$ $d_{wc}=0.1655$, and nuclear export rate $r_{ex}=0.9306$. CLV3-mediated transcriptional regulation fails to maintain *CLV3* expression in L1 and L2 cell layers for lower *WUS* expression rates when *CLV3* is activated within the activation window of WUS concentration (left three columns). It also fails to maintain *CLV3* expression in L1 and L2 cell layers for higher *WUS* expression rates when *CLV3* is activated within the repression window of WUS concentration (middle three columns). The dual regulation with the spatial *CLV3* expression can maintain *CLV3* expression in L1 and L2 cell layers upon both decrease and increase in *WUS* expression rates when *CLV3* is activated within the repression window of WUS concentration (right three columns).

**
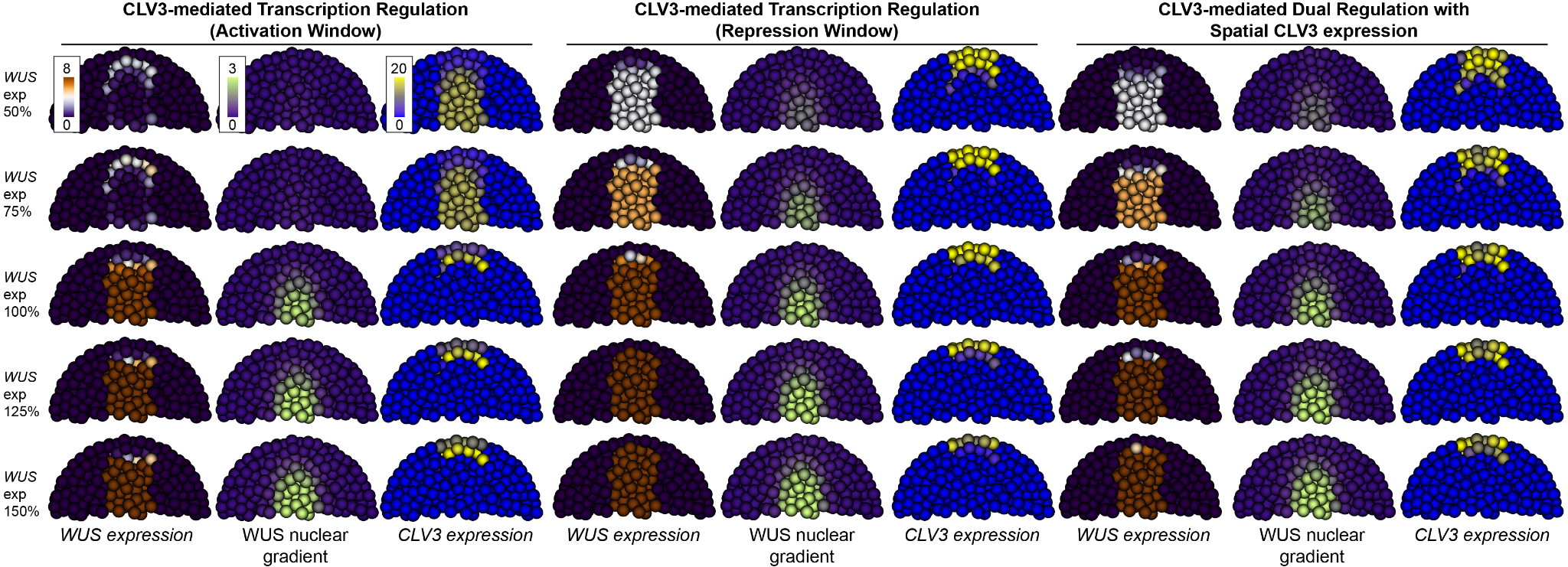
**

**Supplementary Figure 18. Fourth additional parameter set to demonstrate the robustness of the *CLV3* expression domain improved by CLV3-mediated dual regulation with *CLV3* spatial expression.** In this parameter set, WUS mRNA $A_{1}=7.7040$, diffusion rate of WUS $D_{w}=4.1047$, degradation rate of $W_{n}$ $d_{wn}=0.0355$, degradation rate of $W_{c}$ $d_{wc}=7.8439$, and nuclear export rate $r_{ex}=1.1864$. CLV3-mediated transcriptional regulation fails to maintain *CLV3* expression in L1 and L2 cell layers for lower *WUS* expression rates when *CLV3* is activated within the activation window of WUS concentration (left three columns). It also fails to maintain high *CLV3* expression in L1 and L2 cell layers for higher *WUS* expression rates when *CLV3* is activated within the repression window of WUS concentration (middle three columns). The dual regulation with the spatial *CLV3* expression can maintain *CLV3* expression in L1 and L2 cell layers for both reduced and increased *WUS* expression rates when *CLV3* is activated within the repression window of WUS concentration (right three columns).


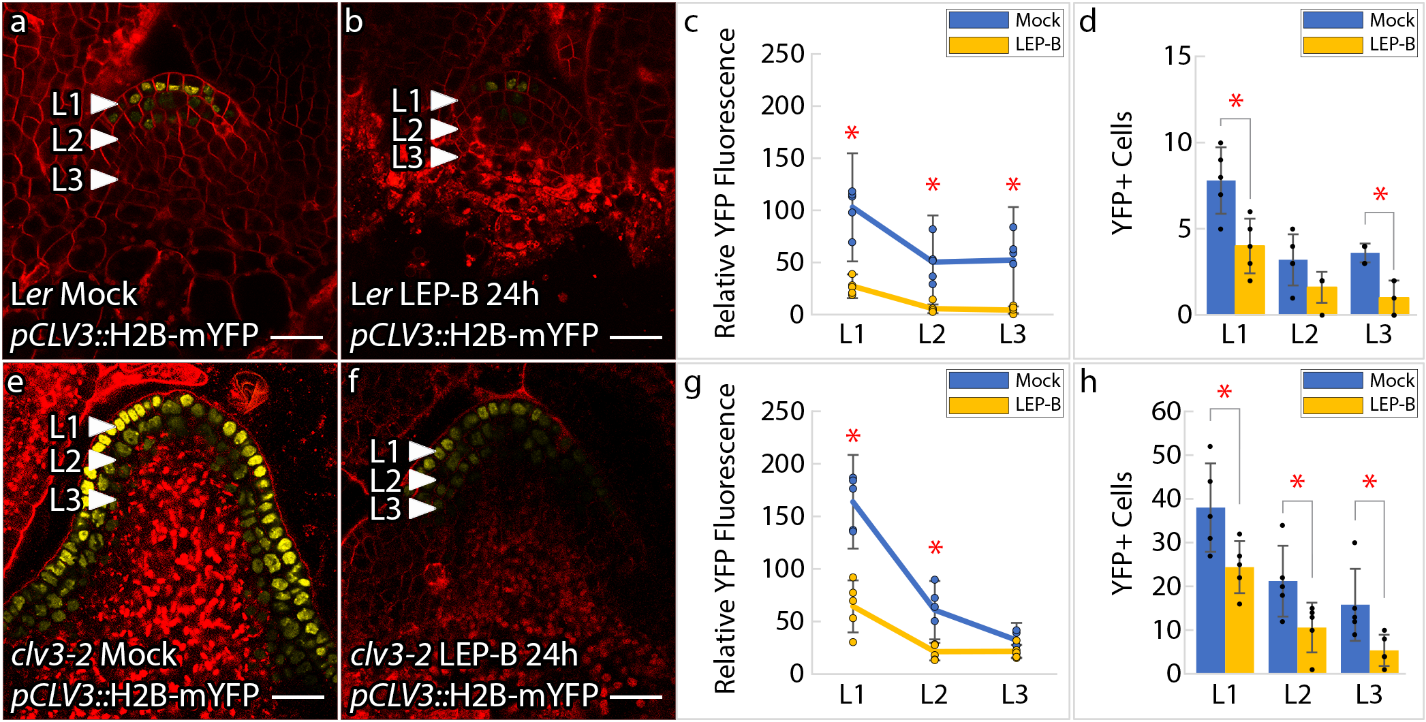


**Supplementary Figure 19. Quantification of *CLV3* expression in response to Leptomycin-B.** (a-b) *pCLV3::*H2B-mYFP reporter in 7-day old wild type plants in response to (a) mock (n=5) or (b) 20nM LEP-B (n=5) treatments for 24 hrs. (c) Quantification of *pCLV3::*H2B-mYFP fluorescence levels (n represents independently treated plants for mock [n=5] and LEP-B [n=5], mean ±s.d.) and (d) the average count of cells that were YFP positive in response to 24 hr LEP-B treatments in wild type from a-b (n represents independently treated plants for mock [n=5] and LEP-B [n=5], mean ±s.d.). (e-f) *pCLV3::*H2B-mYFP reporter in 7-day old *clv3-2* plants in response to (e) mock (n=5) or (f) 20nM LEP-B (n=5) treatments for 24 hrs. (g) Quantification of *pCLV3::*H2B-mYFP fluorescence levels (n represents independently treated plants for mock [n=5] and LEP-B [n=5], mean ±s.d.) and (h) the average count of cells that were YFP positive in response to 24 hr LEP-B treatments in *clv3-2* from e-f (n represents independently treated plants for mock [n=5] and LEP-B [n=5], mean ±s.d.). * represent p.value ≤ 0.05 when compared to mock treatments in the same cell layer (Student’s two-tailed t-test). In all case n represents independently treated plants. Error bars represent the standard deviation. mYFP (yellow) is overlaid on FM4-64 (red) plasma membrane stain. Scale bars = 20 μM.


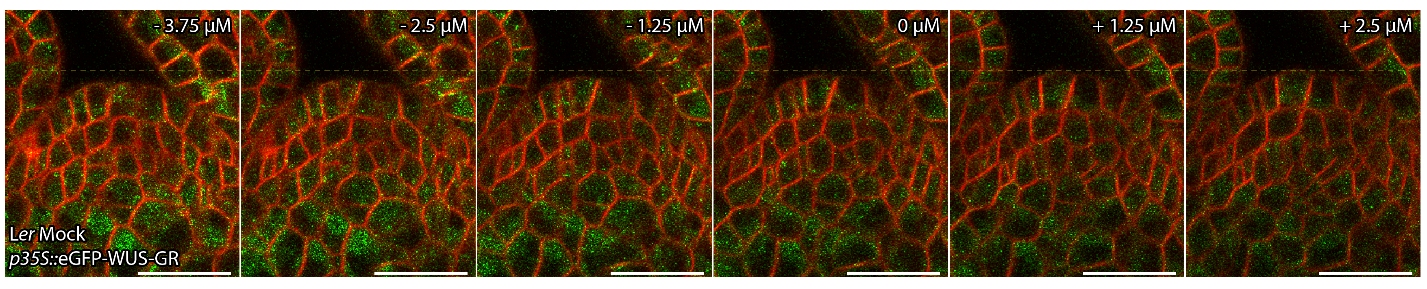


**Supplementary Figure 20. Selection of median of the SAM.** In all images, the median section of each SAM is determined by identifying the region where the SAM apex reaches its peak height (at the 0-2.5 μM slices in the presented series). The yellow dashed line represents the peak of the SAM. eGFP (green) is overlaid on FM4-64 (red) plasma membrane stain. Scale bars = 20 μM

**
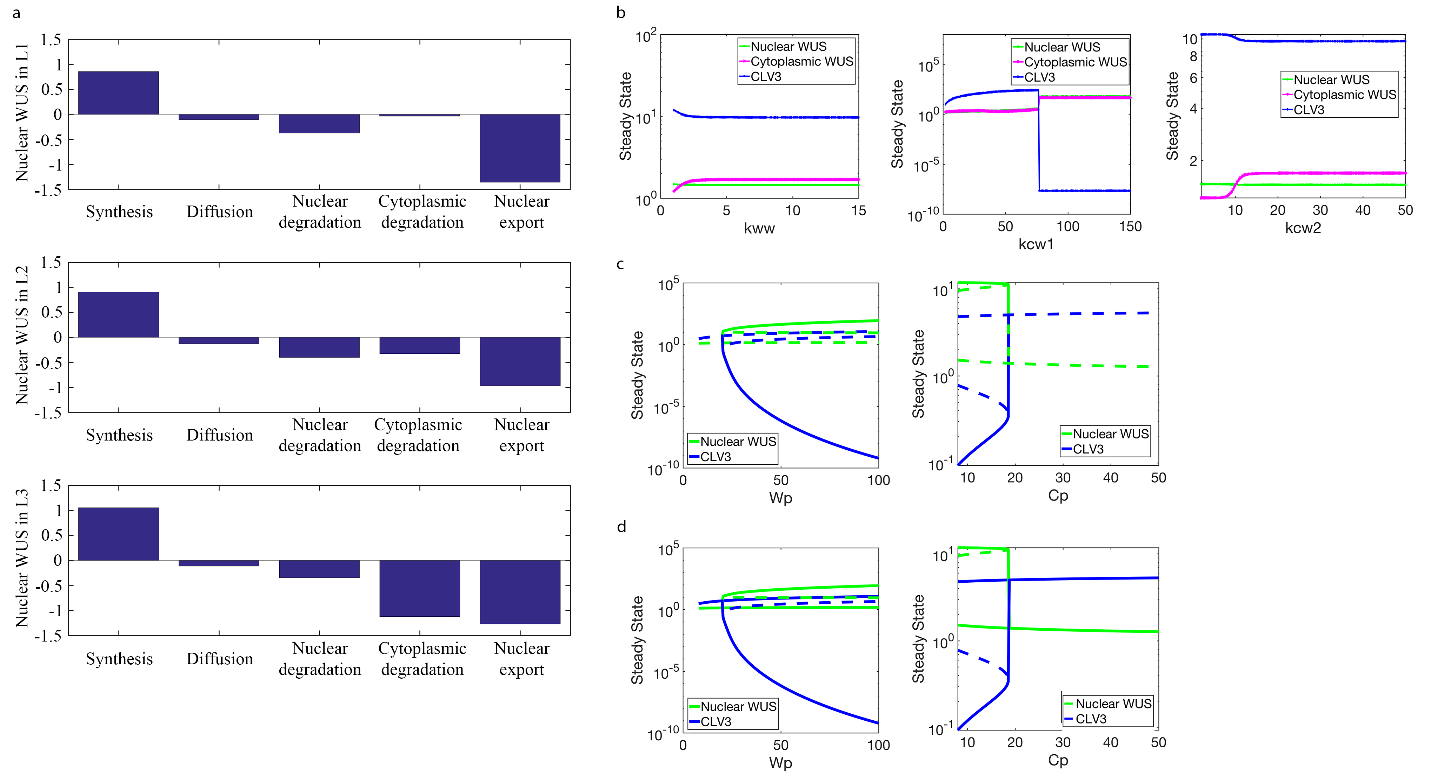
 Supplementary Figure 21. Calibration of the computational model.** (a) Calibration of the basic PDE model. Bar plots of sensitivity analysis of Nuclear WUS in L3, L2 and L1 with respect to *WUS* synthesis rate, WUS diffusion rate, Nuclear WUS degradation rate, Cytoplasmic WUS degradation rate, or Nuclear export rate. Parameters perturbed are shown on the x-axis for the three cell layers and the sensitivity of nuclear WUS protein concentration is shown on the y-axis. (b) Calibration of parameters for feedback regulations. Single parameter perturbation on the stable steady state such that WUS and CLV3 coexist. The x-axis represents perturbed parameters and y-axis represents the steady state concentrations. (c) Steady states of nuclear WUS protein and CLV3 in the ODE model with CLV3 transcriptional regulation only. (d) Steady states of nuclear WUS protein and CLV3 in the ODE model with dual regulations. Three steady states can be obtained for both cases. Stable ones are marked in solid line and unstable ones are marked in dashed line. In the model without post-translational regulation, only the steady state with high nuclear WUS and no CLV3 is stable when $W_{p}$ is too high or $C_{p}$ is too low. In contrast, in the model with dual regulations, there is always a stable steady state such that WUS and CLV3 can coexist over the parameter range explored.


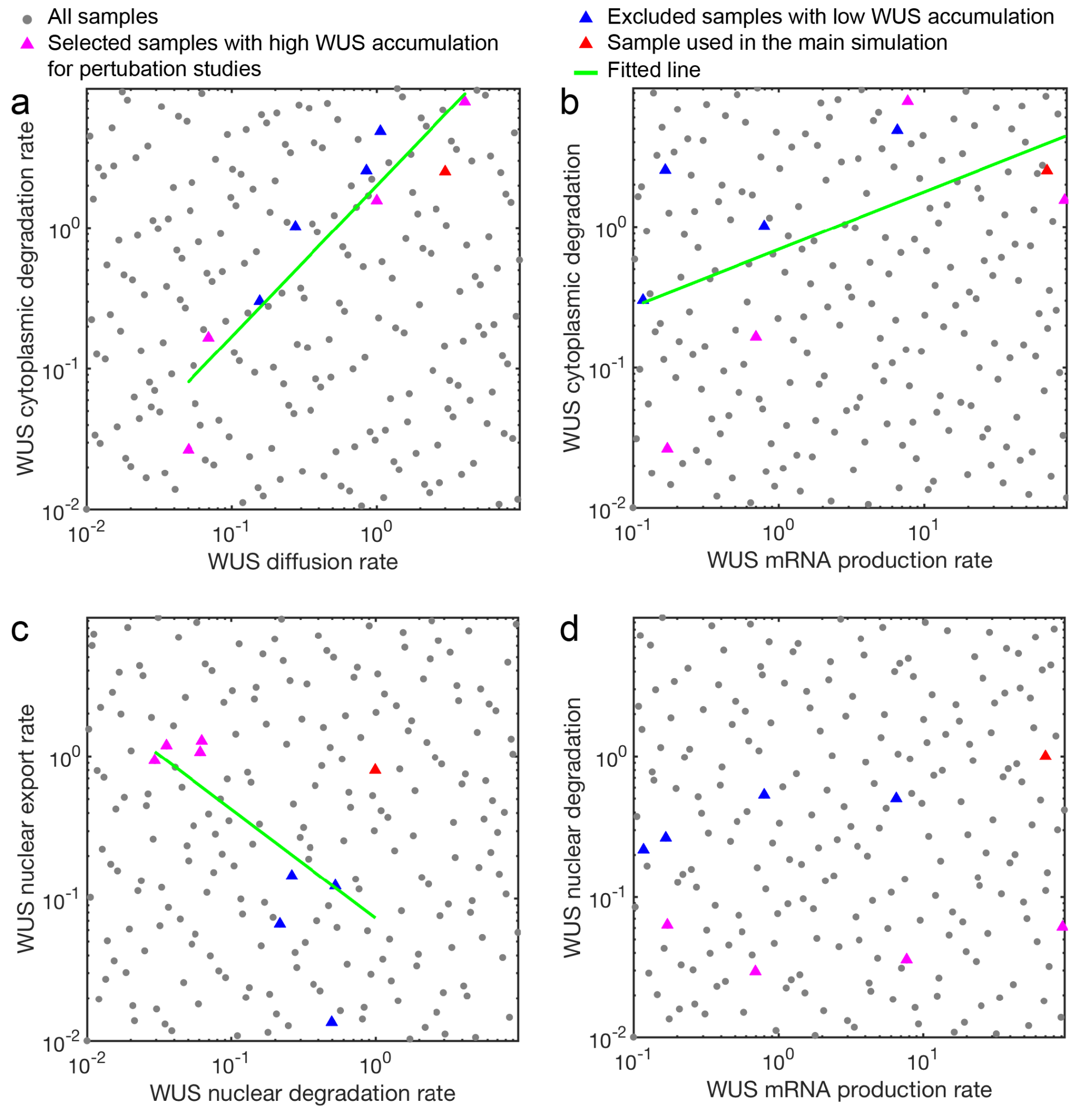


**Supplementary Figure 22. Distribution of parameter samples from global sensitivity analysis using Sobol sampling and samples selected for perturbation studies.** For samples satisfying selection criteria, (a) WUS diffusion rate linearly increases as the cytoplasmic degradation increases under log-log scale; (b) WUS mRNA production rate linearly increases as the cytoplasmic degradation increases under log-log scale; (c) WUS nuclear export rate linearly decreases as the nuclear degradation rate increases under log-log scale; (d) no significant correlation was observed between WUS.

**Supplementary Table 1.**

| **Parameter** | **Definition** | **Value** | **Reference** |
| --- | --- | --- | --- |
| $A_{1}$ | WUS mRNA synthesis rate | 70 | Calibrated in this model |
| $A_{2}$ | CLV3 synthesis rate | 25 | Calibrated in this model |
| $A_{3}$ | Cytokinin synthesis rate | 1 | Calibrated in this model |
| $A_{4}$ | Cytokinin receptor synthesis rate | 1 | Calibrated in this model |
| $D_{w}$ | WUS diffusion rate | 3 | Calibrated in this model |
| $D_{c}$ | CLV3 diffusion rate | 0.1 | Yadav et al. 2013 |
| $D_{ck}$ | Cytokinin ligand diffusion rate | 1 | Calibrated in this model |
| $r_{c}$ | WUS protein synthesis rate | 0.5 | Calibrated in this model |
| $k_{on}$ | Cytokinin ligand-receptor binding rate | 0.5 | Calibrated in this model |
| $W_{\theta}^{1}$ | CLV3 activation peak | 4 | Calibrated in this model |
| $W_{\theta}^{2}$ | WUS-CLV3 inhibition EC50 | 6 | Calibrated in this model |
| $W_{\theta}^{3}$ | WUS-CLV3 activation EC50 | 2 | Calibrated in this model |
| $n_{4}$ | Hill coefficient of WUS activating CLV3 | 10 | Calibrated in this model |
| $d_{c}$ | CLV3 degradation rate | 0.1 | Calibrated in this model |
| $d_{w}$ | WUS mRNA degradation rate | 0.2 | Yadav et al. 2013 |
| $d_{wn}$ | WUS nuclear protein degradation rate | 1 | Calibrated in this model |
| $d_{wc}$ | WUS cytoplasmic protein degradation rate | 2.5 | Calibrated in this model |
| $d_{ck}$ | Cytokinin ligand degradation rate | 0.1 | Calibrated in this model |
| $d_{ckR}$ | Cytokinin receptor degradation rate | 0.1 | Calibrated in this model |
| $d_{CK}$ | Cytokinin complex degradation rate | 0.1 | Calibrated in this model |
| $r_{ex}rex$ | Nuclear export rate | 0.8 | Calibrated in this model |
| $rim$ | Nuclear import rate | 2.4 | Calibrated in this model |
| $k_{cw}^{1}$ | CLV3-WUS inhibition EC50 | 80 | Calibrated in this model |
| $n_{1}$ | Hill coefficient of CLV3 inhibiting WUS | 10 | Calibrated in this model |
| $k_{ck}^{2}$ | Cytokinin-WUS stabilize cytoplasmic protein EC50 | 1 | Calibrated in this model |
| $n_{2}$ | Hill coefficient of Cytokinin stabilizing cytoplasmic protein | 2 | Calibrated in this model |
| $C_{min}$ | Minimal degradation by Cytokinin regulation | 0 | Calibrated in this model |
| $C_{max}$ | Maximal degradation by Cytokinin regulation | 1 | Calibrated in this model |
| $k_{cw}^{3}$ | CLV3-WUS block nuclear export EC50 | 151 | Calibrated in this model |
| $n_{3}$ | Hill coefficient of CLV3 inhibiting nuclear export | 10 | Calibrated in this model |
| $k_{ck}^{1}$ | Cytokinin-WUS block nuclear export EC50 | 1 | Calibrated in this model |
| $n_{5}$ | Hill coefficient of Cytokinin inhibiting nuclear export | 4 | Calibrated in this model |
| $r_{min}$ | Minimal nuclear export rate by Cytokinin regulation | 1 | Calibrated in this model |
| $r_{max}$ | Maximal nuclear export rate by Cytokinin regulation | 2 | Calibrated in this model |
| $k_{cw}^{2}$ | CLV3-WUS block lateral diffusion EC50 | 151 | Calibrated in this model |
| $n_{6}$ | Hill coefficient of CLV3 inhibiting lateral diffusion | 10 | Calibrated in this model |
| $k_{ww}$ | WUS nuclear self-stabilization EC50 | 14 | Calibrated in this model |
| $n_{7}$ | Hill coefficient of WUS nuclear self-stabilization | 4 | Calibrated in this model |
| $d_{min}$ | Minimal degradation of self-stabilization | 1 | Calibrated in this model |
| $d_{max}$ | Maximal degradation of self-stabilization | 3 | Calibrated in this model |
| $r_{w}$ | Radius of WUS expression domain in x-y plane | 3 | Calibrated in this model |
| $L_{w}$ | Maximum height of WUS expression domain in z axis | 8.5 | Calibrated in this model |

Parameters for wild-type simulations and perturbation study.

**Supplementary Table 2.**

| **Parameter** | **Definition** | **Value** | **Reference** |
| --- | --- | --- | --- |
| $r_{ex}rex$ | Nuclear export rate | 2.4 | Calibrated in this model |
| $r_{min}$ | Minimal nuclear export rate by Cytokinin regulation | 0.25 | Calibrated in this model |
| $r_{max}$ | Maximal nuclear export rate by Cytokinin regulation | 2 | Calibrated in this model |

Parameters for perturbation study.

**Supplementary Table 3.**

| **Parameter** | **Definition** | **Value** | **Range(+/- 75%)** |
| --- | --- | --- | --- |
| $A_{1}$ | WUS mRNA synthesis rate | 10 | [2.5, 17.5] |
| $D_{w}$ | WUS diffusion rate | 1 | [0.25, 1.75] |
| $d_{wn}$ | WUS nuclear protein degradation rate | 0.12 | [0.03, 0.21] |
| $d_{wc}$ | WUS cytoplasmic protein degradation rate | 1 | [0.25, 1.75] |
| $r_{ex}rex$ | Maximal nuclear export rate | 0.5 | [0.125,0.875] |

Parameters for sensitivity analysis.

**Supplementary Table 4.**

| **Peptide** | **Peptide Name** | **Sequence** |
| --- | --- | --- |
| Scrambled CLV3 peptide | sCLV3 | PPTRGLSHHPVD |
| Bioactive CLV3 peptide | MCLV3 | RTVPSGPDPLHH |

Peptides used in this study.

**Supplementary Table 5.**

| **Gene** | **Primer Name** | **Sequence** |
| --- | --- | --- |
| WUS | WUS-Fwd | ATCATGCAAGCTCAGGTACTGAATGT |
|  | WUS-Rev | GAGCTTTAATCCCGAGCGACACCGG |
| UBIQUITIN10 | UBQ10-Fwd | GATCTTTGCCGGAAAACAATTGGAGGA |
|  | UBQ10-Rev | CGACTTGTCATTAGAAAGAAAGAGATACA |
| AtXPO1a | XPO1a fwd | ATGGCGGCTGAGAAGTTAAGGG |
|  | XPO1a rev | TTATGAGTCCACCATCTCGTCTTG |
| AtXPO1b | XPO1b fwd | ATGGCTGCCGAGAAGTTAAGA |
|  | XPO1b rev | TTAAGAATCGGCCATGTCGTCTTG |
| WUS EAR-like domain mutation | 5’-EARLM fwd | CACGTGTGAACTAGGCCTGC |
|  | 5’-EARLM rev | CCTCAGCAGAAGCGCAAGG |

Primers used in this study.

**Supplementary Table 6.**

| **Parameter** | **Definition** | **First** | **Second** | **Third** | **Fourth** | **Range** |
| --- | --- | --- | --- | --- | --- | --- |
| $A_{1}$ | WUS mRNA synthesis rate | 0.1715 | 92.224 | 0.6978 | 7.704 | [0.1, 100] |
| $D_{w}$ | WUS diffusion rate | 0.0505 | 1.009 | 0.0698 | 4.1047 | [0.01, 10] |
| $d_{wn}$ | WUS nuclear protein degradation rate | 0.0626 | 0.061 | 0.0294 | 0.0355 | [0.01, 10] |
| $d_{wc}$ | WUS cytoplasmic protein degradation rate | 0.0264 | 1.5538 | 0.1655 | 7.8439 | [0.01, 10] |
| $rr_{ex}ex$ | Maximal nuclear export rate | 1.2864 | 1.065 | 0.9306 | 1.1864 | [0.01, 10] |

Additional parameter sets from global sensitivity analysis.

**Supplementary Movie 1.**

The *CLV3* expression profile reaching the steady state in the model simulation.

**Supplementary Movie 2.**

The nuclear WUS protein gradient reaching the steady state in the model simulation.
