# Supplementary material for: CLAVATA3 mediated simultaneous control of transcriptional and post-translational processes provides robustness to the WUSCHEL gradient
Source: Nat Commun. 2021 Nov 4;12:6361. doi: 10.1038/s41467-021-26586-0 (PMC8569176; doi:10.1038/s41467-021-26586-0)
Supplement: Supplementary file 3 — Description of Additional Supplementary Files [file 41467_2021_26586_MOESM3_ESM.pdf]

Description of Additional Supplementary Files

File name: Supplementary Movie 1

Description: The CLV3 expression profile reaching the steady state in the model simulation.

File name: Supplementary Movie 2

Description: The nuclear WUS protein gradient reaching the steady state in the model simulation.
